# Incidence and risk factors for epidural morphine induced pruritus in parturients receiving cesarean section: A prospective multicenter observational study

**DOI:** 10.1097/MD.0000000000017366

**Published:** 2019-10-04

**Authors:** Xiao Tan, Le Shen, Lin Wang, Labaciren, Yuelun Zhang, Xiuhua Zhang, Yuguang Huang

**Affiliations:** a Department of Anesthesiology, Peking Union Medical College Hospital, Dongcheng District; b Department of Anesthesiology, Beijing Obstetrics and Gynecology Hospital, Capital Medical University, Chaoyang District, Beijing; c Department of Anesthesiology, Tibet Autonomous Region People's Hospital, Lhasa; d Central Research Laboratory, Peking Union Medical College Hospital, Dongcheng District, Beijing, China.

**Keywords:** adverse effects, allergy, epidural morphine, pruritus, risk factors

## Abstract

This study is designed to clarify the incidence of epidural morphine induced pruritus (EMIP) in Chinese Han and Tibetan parturients after cesarean section (CS) and to identify the correlated risk factors.

This is a prospective multicenter observational study. Follow-up evaluations were performed at 3 hours, 6 hours, 12 hours, 24 hours, and 48 hours after morphine administration. The primary outcome was EMIP incidence. Other details were also recorded for risk factor screening.

Totally 284 parturients receiving CS were enrolled and 247 completed the study. The overall incidence of EMIP was 18.6% (46 in 247). The onset of pruritus was 5.6 ± 4.8 hours (mean ± SD) after morphine administration and the duration of pruritus was 14.0 ± 8.8 hours. Logistic regression models was built with 5 variables, history of allergy, serotonin receptor antagonist administration, I.V. fentanyl administration, epidural morphine volume and VAS pain score. Results of showed that 2 of the variables, history of allergy (*P* < .001) and serotonin receptor antagonist (*P* < .05), were significantly correlated with incidence of EMIP.

In conclusion, EMIP incidence in our study was 18.6%. Positive medical history of allergy and not using serotonin receptor antagonist were potential risk factors of EMIP development.

**Trial registration:** ChiCTR-OPC-17012345

## 1 Introduction

Epidural morphine provides prolonged and effective postoperative analgesia, and it is commonly used for analgesia after cesarean section (CS).^[1]^ While spinal morphine is a common method for post-cesarean analgesia in European and Americas, epidural morphine remains a routine technique in mainland China. Among the side effects of neuraxial morphine, pruritus is one of the most troublesome.^[2,3]^ Mild pruritus is sometimes ignored, but sometimes severe itch after epidural morphine significantly affect patients’ daily life as well as recovery.

Much remains unknown about pruritus. Pregnant women seem to be more susceptible to neuraxial opioid than other populations, perhaps due to an interaction of estrogen with opioid receptors.^[3]^ The incidence of pruritus after intrathecal morphine administration is reported to be as high as 85% in parturients,^[4,5]^ and that by epidural morphine varies between 20% and 73% in the literature.^[6,7]^ The large variation may be due to the different definitions of morphine induced pruritus in different studies, and it happens that sometimes mild itching is neglected in clinical observations. Therefore, this study is aiming to identify the incidence of epidural morphine-induced pruritus (EMIP) in mainland China, where epidural morphine remains a routine practice after CS in most institutes. Furthermore, potential risk factors for EMIP are also noted. Since data on the management of pruritus are limited,^[2]^ the identification of correlated risk factors holds great clinical significance for better guiding EMIP treatment.

## 2 Materials and methods

### 2.1 Study population

This is a prospective multicenter observational study, registered on Chinese Clinical Trial Registry (http://www.chictr.org.cn/index.aspx, registration number ChiCTR-OPC-17012345). This study underwent ethics review and received approval from the Peking Union Medical College Hospital Ethics Review Board. Written informed consent was achieved in each participant before enrollment. Parturients receiving elective CS were selected as our study population for the following reasons. Firstly, previous studies have confirmed that parturients are more likely to develop EMIP, perhaps due to an interaction of estrogen with opioid receptors.^[3]^ Secondly, epidural morphine is routinely administered after CS in most institutes in China, while it is not administered in other types of operations. Three medical centers in China (Peking Union Medical College Hospital, Beijing Obstetrics and Gynecology Hospital, Tibet Autonomous Region People's Hospital) were involved. All the participants were ASA II (According to ASA Physical Status Classification System, pregnancy should be classified into ASA II).^[8]^ Each received combined spinal and epidural anesthesia (CSEA) or epidural anesthesia (EA). As in our institute, morphine was not allowed to be administered intrathecally, thus those receiving single spinal anesthesia without an epidural catheter cannot be included in this study. Exclusive criteria were as follows:

1. Morphine not administered for postoperative analgesia;
2. Any existing itchy skin diseases;
3. ASA III or higher;
4. Urgent CS under general anesthesia;
5. Back to ICU after delivery;

### 2.2 Study protocol

Patients’ basic information including age, gravidity and parity, gestational age, body mass index (BMI), medical history of pregnancy, allergy, drug administration, alcohol and tobacco, etc was collected before surgery. All the information was derived from the patients’ medical records. Each patient was instructed about the study and particularly about the use of the Visual Analogue Scale (VAS) for pain as well as pruritus during the interview 1 day before surgery. Written informed consent was obtained during preoperative interview. On the surgical day, patients were routinely monitored with continuous electrocardiography, pulse oximetry, and intermittent noninvasive blood pressure. Intravertebral anesthesia (CSEA or EA) was performed in each patient. As mentioned above, patients receiving single spinal anesthesia were not involved in this study. For CSEA, spinal anesthetic (usually 0.5% bupivacaine or 0.5% ropivacaine) was administered to initiate anesthesia and an epidural catheter was inserted for anesthesia maintaining and postoperative analgesia. For EA, a mixture of 1% lidocaine and 0.5% ropivacaine was given epidurally. Once a satisfactory sensory block (T4 ± 2 segments) was obtained by loss of sensation to cold, a standard CS was performed. As for an observational study, no intervention was taken in the type or dosage of any drug administered.

Epidural morphine was administered after delivery and before the end of the operation, and the exact timing of morphine administration was recorded, so as the morphine dosage and volume. As our routine clinical practice, epidural morphine was usually administered in a dosage of 2 to 3 mg, which was comparable to similar studies and was about 10 times to other studies using intrathecal morphine.^[9,10]^ After delivery of the baby, other intravenous medications were given for sedation (e.g., midazolam), analgesia (e.g., fentanyl) or prevention of PONV (e.g., ondansetron). Every drug administration was recorded according to patients’ anesthesia records. Vasoactive drugs (e.g., ephedrine and phenylephrine) were not recorded in this CRF. Furthermore, amounts of fluids infusion, blood loss and urine output were also recorded. The epidural catheter was removed immediately after the surgery and patients were sent back to ward.

The primary outcome of this study was the incidence of EMIP. According to literature, typical sites of EMIP included the face, trunk, upper limbs, lower limbs, and occasionally even the whole body.^[6]^ Thus, we define EMIP in our study as follows:

1. Morphine was administered epidurally;
2. Pruritus developed within 48 hours after CS;
3. No pruritus before surgery;
4. Pruritus caused by other factors was excluded.

The pruritus onset and resolution time, duration, sites and intensity were noted. Intensity of pruritus was evaluated by VAS.^[11]^ Secondary outcome were rest and exercise VAS pain scores. Follow-ups were performed at 3 hours, 6 hours, 12 hours, 24 hours, and 48 hours after epidural morphine administration The timing of follow-ups was designed according to other similar study protocols and the final follow-up was set later than other studies.^[12,9]^ For patients with EMIP, clinical observation continued until the end of itching, which could be possibly longer than 48 hours postoperatively.

According to literature, variables associated with EMIP include morphine dosage,^[10]^ serotonin receptor antagonist,^[13–15]^ VAS pain score,^[3,16]^ and possibly other unknown factors. All the variables above were included in our observation. Other potential variables and confounding factors included: age, BMI, past births, ethnic group, past history of allergy, history of tobacco and alcohol, IV fentanyl administration. As an observational study, we collect as comprehensive clinical data as possible. Other details as fluid infusion, urine output and estimated blood loss were also collected.

### 2.3 Sample size

Sample size estimation was based on the sample size estimating equation for investigating one proportion with confidence interval. Based on our previous experience in our institute, the estimated incidence of EMIP was set 20%. Two hundred sixty-five patients were needed to achieve an expected proportion of 20% with a total width of 2-sided 95% confidence interval of 0.1.

### 2.4 Statistical analysis

Information of each patient was collected using a case report form (CRF), including pre-, intra-, and post-operative assessments. Categorical variables were expressed as frequencies and percentages, while continuous variables were summarized as means ± SD in the descriptive analysis. Univariate analysis was performed to screen the potential risk factors for further analysis. Chi-square test and *t* test were performed for categorical variables and continuous variables, respectively. Bonferroni adjustment to *P* value was used when comparing variables with possibility of multiple comparisons. Variables in multivariate logistic regression were screened based on clinical experience and results of univariate analysis. First, we identified factors that were related to EMIP according to literature and clinical experience. These variables were forced into the regression model, whether or not there was statistical significance in univariate analysis. In addition, variables with *P* < .2 in univariate analysis were also included in the regression model. Binary Logistic regression was chosen as multivariate model to explore potential risk factors. Hosmer and Lemeshow test was used to estimate the goodness-of-fit of Logistic regression model. If *P* < .1, method of Backward Stepwise (Wald) was used to build a regression model that meets the goodness-of-fit requirement. Interaction effects between variables were also considered and tested. ROC curve was plotted and the area under the curve (AUC) was calculated to test the power of the model. Statistical analyses were conducted using SPSS Statistics 22.0 (IBM, USA) and two-sided *P* < .05 was considered statistically significant.

## 3 Results

### 3.1 Baseline information of participants

**Figure 1:**
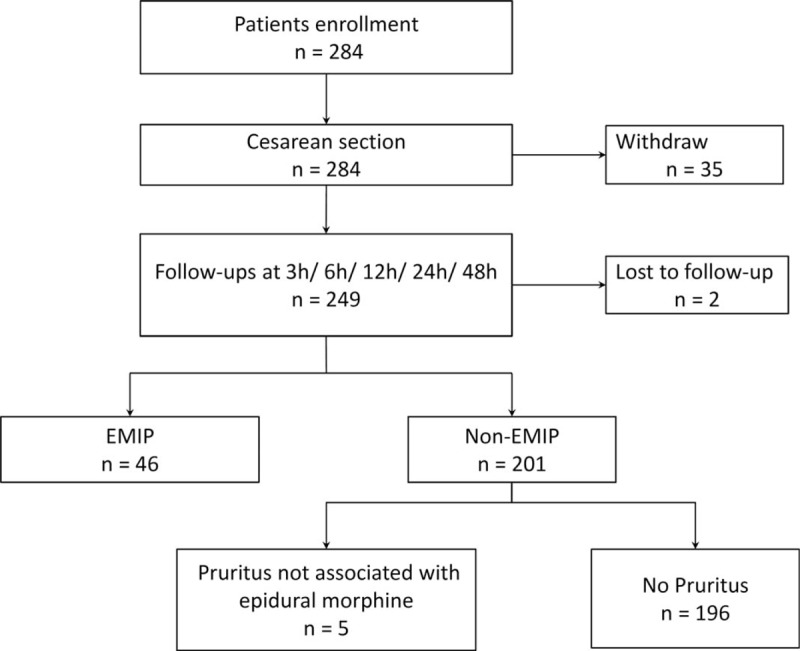
Study flow chart.

From January 2017 to October 2018, 284 parturients were enrolled from 3 institutes in mainland China (194 from Peking Union Medical College Hospital, 73 from Beijing Obstetrics and Gynecology Hospital, and 17 from Tibet Autonomous Region People's Hospital). All the parturients we approached agreed to be enrolled in this study. Written informed consent was obtained during pre-operative interview. As is shown in Figure 1, among the 284 participants, n = 37 were excluded or lost to follow-up. Morphine was not administered in n = 35 (because epidural catheter was not smoothly placed, thus morphine could not be administered epidurally). n = 2 parturients were lost to follow-up because they were sent back home within 24 hours after surgery. Therefore, n = 247 finally completed the study. Among the parturients enrolled, there are 17 Tibetans and 230 Han Chinese. The average age, gestation week, BMI of the participants who completed the study were shown in Table 1.

**Table 1:**
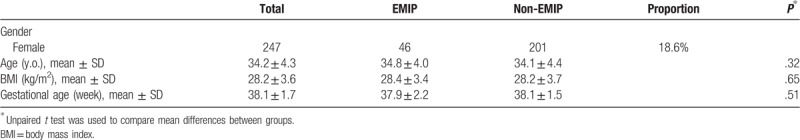
Demographics of patients enrolled.

### 3.2 Incidence of EMIP

There were n = 46 patients developed EMIP after surgery, with an incidence of 18.6%. Statistical analysis showed that patients with or without EMIP were not significantly different in terms of age, gestational age, BMI (See Table 1). The 49 patients who developed EMIP mainly had pruritus of the face, trunk, upper limbs, lower limbs, or throughout the body. The itching emerged at 5.6 ± 4.8 (0.3 hour to 20.0 hours) after morphine administration and lasted for 14.0 ± 8.8 hours (3 hours to 36 hours). Most patients developed mild or moderate itching, but there were also n = 4 patients who experienced severe itching (affecting sleep and daily life). For those who developed itching, decision of whether or not to treat the symptom was made by the obstetricians. Clinical observations found that no special antipruritic drugs were given, and the symptom of itching was obviously relieved in our next follow-up. There were also n = 6 patients who developed itching which was clearly derived from aseptic dressings, medical tapes or clothes, thus were excluded from positive cases.

### 3.3 Pain and pruritus VAS scores

**Figure 2:**
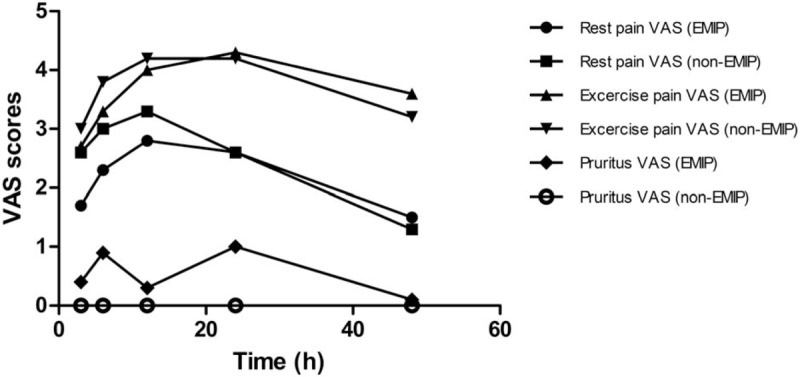
Pain VAS score versus time. Six curves are showing the pain VAS score (rest and exercise) and pruritus VAS score of EMIP and non-EMIP groups with time.

VAS scores of pain as well as pruritus were evaluated by each participant during follow-ups. Pain VAS included rest and exercise scores. Data were summarized as mean ± SD and VAS-time curves were plotted (Fig. 2 and Table 2). In either group, pain scores were relatively low at 3 hours, and increased gradually afterwards. The scores peaked at around 12 to 24 hours and gradually decreased at 48 hours, the last follow-up. Comparing the 2 groups, rest and exercise VAS pain scores of the EMIP group were always lower than non-EMIP group at 3 hours, 6 hours, and 12 hours, and the scores at 24 hours and 48 hours went closer. Bonferroni adjustment was used in statistical analysis to avoid multiple comparisons. Since there are 5 time points involved in comparing VAS scores, statistically significant threshold was 0.05/5 = 0.01. Therefore, statistics showed no significant difference in any time point between EMIP and non-EMIP groups.

**Table 2:**
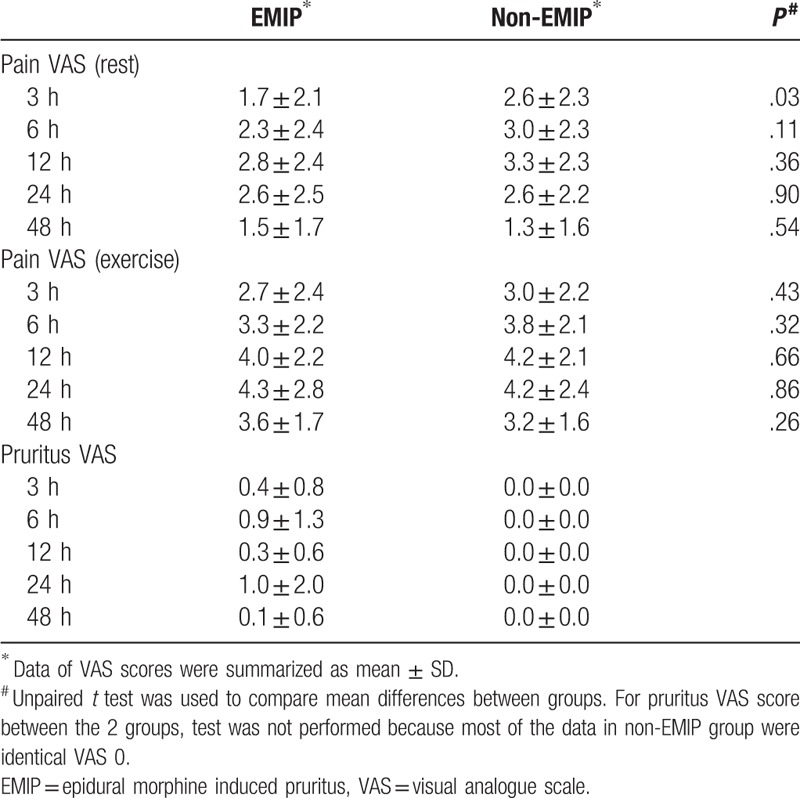
Pain VAS score (rest and exercise) and pruritus VAS score.

### 3.4 Multivariate regression model

Among the different clinical features of these parturients, we screened the following 9 variables as potential risk factors for univariate analysis: ethnic group, serotonin (5-HT3) receptor antagonist, I.V. fentanyl, medical history of allergy, epidural morphine volume, age, VAS pain score, past births and BMI. Except for VAS pain score, all the variables were categorical. Results of univariate analysis of each variable are listed in Table 3. Some details of these factors are listed as follows. For serotonin receptor antagonists, drugs administered included several types: ondansetron (2 ml/4 mg) 4 mg or 8 mg IV, granisetron (3 ml/3 mg) 3 mg IV, or tropisetron (5 ml/5 mg) 5 mg IV. Those who received any kind of serotonin receptor antagonist were classified as 1 group, while patients who did not receive any kind of this drug were grouped as the other. For morphine administration, Epidural morphine 2 mg was administered in each patient. Morphine was diluted by NS to either 2 ml (n = 146) or 10 ml (n = 101). For I.V. fentanyl, some of the patients (n = 24) received I.V. fentanyl 50 μg for better analgesia, while the others did not receive fentanyl. None of the patients received other doses of fentanyl.

**Table 3:**
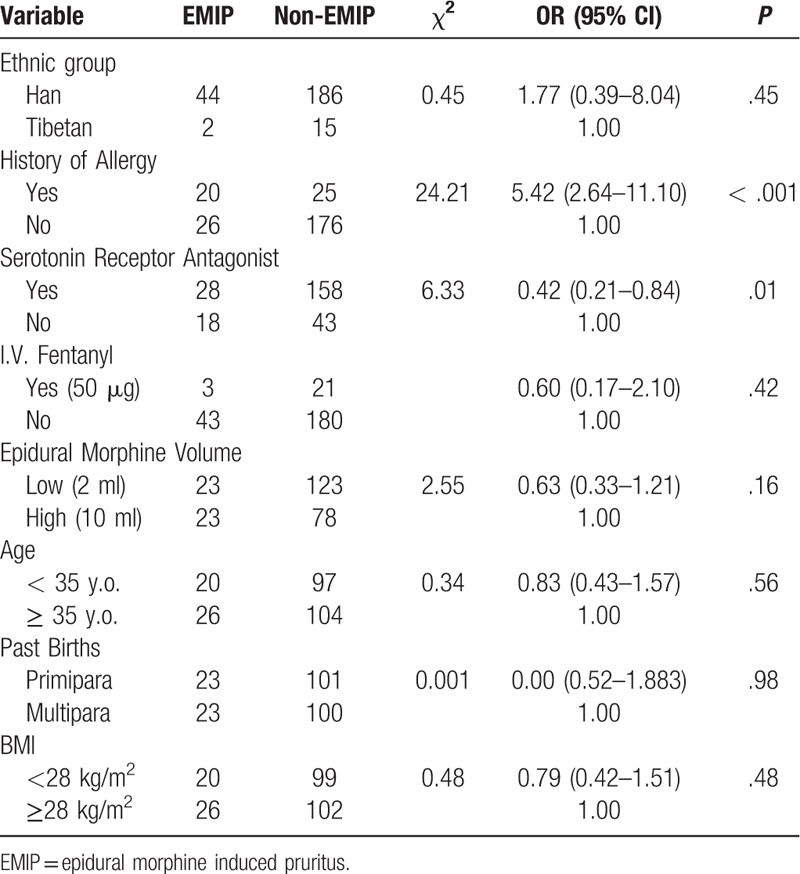
Potential risk factors of EMIP (univariate analysis).

For factor screening of multivariate analysis, we first relied on existed study and clinical experience. Previous literature indicated that serotonin receptor antagonist,^[13]^ I.V. fentanyl,^[14]^ VAS pain score^[3]^ may affect the development of EMIP. Thus, they were selected directly into multivariate regression. Second, we screened factors according to results of univariate analysis. Three of the variates (history of allergy, epidural morphine volume, serotonin receptor antagonist) had a *P* < .2 in univariate analysis. Since serotonin receptor antagonist had already selected, we here further recruited 2 more variables, history of allergy and epidural morphine volume, into multivariate analysis. Therefore, a multivariate model of 5 variables was set, including: serotonin receptor antagonist, I.V. fentanyl, VAS pain score, history of allergy, epidural morphine volume. VAS score is the only continuous factor in this regression model. Since none of the VAS scores showed statistical significance after Bonferroni adjustment (Fig. 2 and Table 2), we chose the time point with relatively the most obvious difference in visual observation. Thus 3 hours VAS pain score at rest was selected into multivariate regression model.

### 3.5 Validity of regression model

**Figure 3:**
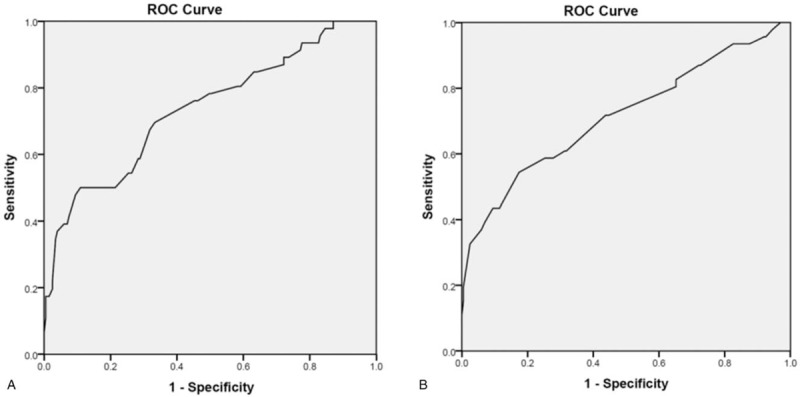
ROC curves of the 2 logistic regression models. The model established with Enter (A) and Wald (B) method has an AUC of 0.735 and 0.715, respectively.

We established a logistic regression model with 5 variables, in which VAS pain score is continuous and the others are categorical variables. According to methodology, we used both Enter and Backward Stepwise (Wald) to test the robustness of this model. The 2 models showed similar results (Tables 4 and 5). Furthermore, interaction effect between variables was considered and tested. We established another Logistic model of 15 variables, including 5 original and 10 new variables formed by their combinations. Results showed none the interaction items had any statistical significance, which indicated there was no significant interaction effect. Finally, ROC curve of the 2 Logistic models were plotted and AUC were calculated. Logistic models of Enter and Wald method had an AUC of 0.735 and 0.715, respectively (Fig. 3). In summary, we consider our multivariate models of this study were stable and results were reliable.

**Table 4:**
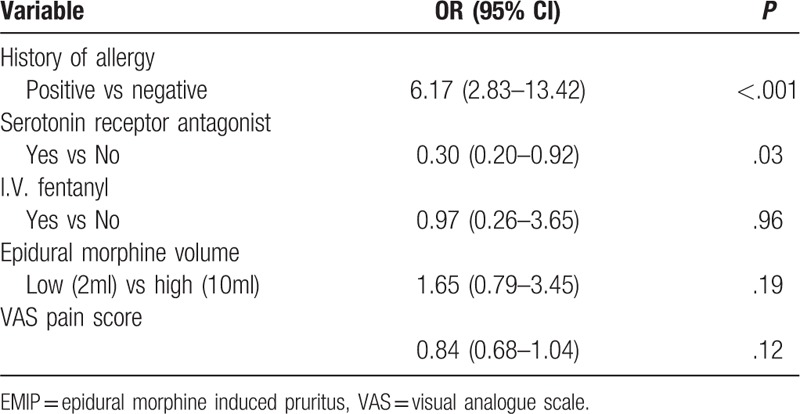
Potential risk factors of EMIP (multivariate logistic regression, ENTER method).

**Table 5:**
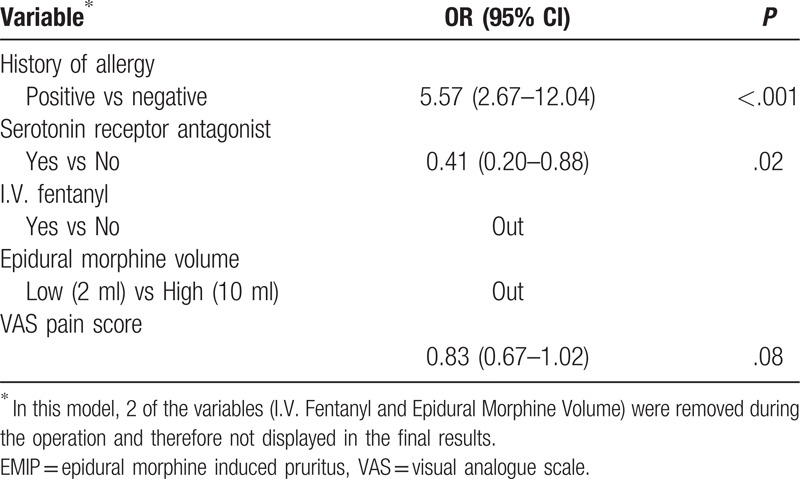
Potential risk factors of EMIP (multivariate logistic regression, WALD method).

### 3.6 Risk factors of EMIP

According to the multivariate regression models, we came to the results that history of allergy had a strong correlation with EMIP (*P* < .001). Also, serotonin receptor antagonist was correlated to EMIP development. In contrast, other variables (I.V. fentanyl, epidural morphine volume, VAS pain score, etc) failed to show statistical significance (Tables 4 and 5).

## 4 Discussion

### 4.1 Incidence of EMIP in Asian population

In this study, we found 46 of 247 parturients developed typical symptom of EMIP, with a proportion of 18.6%. Significant correlations between medical history of allergy, serotonin receptor antagonist and the incidence of EMIP were also found as potential risk factors. In this study, all the participants enrolled were Asian, and n = 230 were derived from Han Chinese and the other n = 17 were Tibetan. Though the number of Tibetan is limited, this is perhaps the first report of Tibetans’ EMIP after CS, laying foundations for further study of Chinese Tibetans in the future. Compared to similar studies from other institutes, there are also studies reporting similar incidence of pruritus. Singh SI reported EMIP incidence was 20% for 1.5 mg and 50% for 3 mg epidural morphine and Mo Y reported intrathecal morphine induced pruritus was 27.5%.^[5,17]^ The 2 studies above were both based on Asian populations. It is unclear whether people from different ethnic groups may develop different EMIP incidence, but some clues are supporting this hypothesis. There are studies reporting relatively lower pruritus rates in Asian population while most European studies reported much higher incidence.^[10,9,18]^ Moreover, there are studies stating that different gene sequences may lead to different pruritus rates.^[19]^ These are all leading us to further investigate if ethnic group is a risk factor of pruritus, which can be future study directions. Overall, as the number of patients enrolled is equivalent to the expected sample size, we consider our findings can be applied in Asian populations.

### 4.2 EMIP and allergy

One of our key findings is a significant correlation between medical history of allergy and the incidence of EMIP (Tables 3 and 4). Results indicated that patients with allergies were more prone to develop EMIP. In our study, there were n = 45 reporting previous allergy to various foods or drugs. Allergens included seafood, alcohol, buckwheat, penicillin, cephalosporin, sulfonamide, erythromycin, etc. Allergens and opioids can both develop pruritus in patients, but whether or not there are common signaling pathway is still unclear. Although the possible mechanism is still unclear, experiments have shown that the incidence of pruritus is related to the diversity of certain molecules. Tsai FF found that the mu opioid receptor gene (OPRM1) polymorphism was associated with the incidence of EMIP and that the recessive allele G in A118G might have a protective effect in cases of severe pruritus.^[19,20]^ Our finding of allergy as a strong risk factor of EMIP lays foundation to further research regarding the prevention and treatment of pruritus.

### 4.3 EMIP and serotonin receptor antagonists

Another finding of this study is that serotonin receptor antagonist administration is also correlated with EMIP incidence. According to results of our study, not using serotonin receptor antagonist is a potential risk factor of EMIP. As a common clinical practice, serotonin receptor antagonists were administered mainly for prevention of PONV. Type of serotonin receptor antagonists included ondansetron, granisetron and tropisetron, depending on different clinical practice in different institutes. Meanwhile, as the administration of serotonin receptor antagonists was not a routine practice in every institute, there were still a small proportion of patients who did not receive any kind of serotonin receptor antagonists. Results showed serotonin receptor antagonist was also a risk factor of EMIP. Some findings are supporting the role serotonin receptor plays in the development of pruritus. A newly published study demonstrates that serum serotonin level increased significantly in the post-cesarean patients, suggesting a role of serotonin in the genesis of intrathecal morphine-induced pruritus.^[15]^ However, literature review shows controversy regarding serotonin receptor antagonists and EMIP. Meta-analyses indicates that the prophylactic use of ondansetron does not reduce the incidence of EMIP but could significantly relieve the severity of pruritus and effectively reduce the amount of drug rescue needed.^[13,14]^ The association of serotonin receptor antagonists and EMIP may be further studied in the future.

### 4.4 EMIP and other potential factors

In this study, we were also intended to find other risk factors, including VAS pain scores, morphine dilution and volume, I.V. fentanyl, age, past births, BMI, etc. However, statistics showed none of them had significant correlation with EMIP incidence.

For VAS pain scores, there are theories indicating that pain and itch are sharing a population of sensory neurons.^[3,16]^ Activation of one signal pathway may inhibit the other. Our study also came to similar findings: VAS pain scores were lower in EMIP group than non-EMIP group (Fig. 2 and Table 2). Perhaps due to our limited sample size, only the VAS pain scores at 3 hours after morphine administration were statistically different. At least our result showed a tendency of the relationship between EMIP development and the intensity of pain. Future studies can be designed to investigate the relationship between pain and itch and its molecular pathways.

As for epidural morphine, all the participants in this study received 2 mg morphine, and the volume as well as dilution of morphine were divided into 2 groups (2 ml and 10 ml). A recent published meta-analysis found pruritus rate was dose-dependent according to different regimen of epidural morphine.^[10]^ However, no hypothesis of whether the volume or concentration of morphine was related to EMIP occurrence was made in that study. The 2 regimens of epidural morphine in our study make little difference in EMIP development.

For intra-operative opioids administration, it may also lead to pruritus after the surgery, thus it is included in our analysis. In our study, only a minority received intra-operative opioids (n = 24) and all the people received identical dosage, fentanyl 50 μg. Result showing no significance between intra-operative fentanyl and EMIP was perhaps due to the limited sample size. There is a limitation in our study that we did not include rescue drugs of analgesia after CS, which can be a confounder of EMIP. However, all the institutes in our study hardly use opioids for analgesia of post-cesarean patients. Common rescue drugs are NSAIDs as ibuprofen, etc. We consider the effect of NSAIDs on EMIP is relatively minor compared to opioids, and that will not have a significant impact on our results.

### 4.5 Limitations

As an observational study, the sample size was consistent with our expectation, and statistical methods effectively reduced bias. Our results can be generalized to reflect EMIP in Asian parturients. Though, expanding the sample size can further reduce the residual confounding effect. The results are only preliminary exploration of EMIP and its correlated factors.

## 5 Conclusion

Through this prospective multicenter observational study recruiting over 200 parturients, we found an EMIP incidence of 18.6%. By establishing a stable multivariate regression model, it was seen that positive medical history of allergy and not using serotonin receptor antagonist were potential risk factors of EMIP development.

## Author contributions

**Data curation:** Xiao Tan, Lin Wang, Labaciren, Yuelun Zhang.

**Investigation:** Le Shen, Xiuhua Zhang, Yuguang Huang.

**Methodology:** Yuelun Zhang.

**Project administration:** Xiao Tan, Le Shen, Xiuhua Zhang.

**Resources:** Lin Wang, Labaciren, Yuguang Huang.

**Software:** Yuelun Zhang.

**Supervision:** Le Shen, Yuguang Huang.

**Validation:** Yuelun Zhang.

**Writing – original draft:** Xiao Tan.

**Writing – review & editing:** Le Shen.

Le Shen orcid: 0000-0002-2563-0012.

## References

[1] Macias MN Hall TG Ostlund J Extended-release epidural morphine and postoperative nausea or vomiting. Am J Health Syst Pharm 2008;65:200. 18216000 10.2146/ajhp070410

[2] Dominguez JE Habib AS Prophylaxis and treatment of the side-effects of neuraxial morphine analgesia following cesarean delivery. Curr Opin Anaesthesiol 2013;26:288–95. 23563797 10.1097/ACO.0b013e328360b086

[3] Kumar K Singh SI Neuraxial opioid-induced pruritus: an update. J Anaesthesiol Clin Pharmacol 2013;29:303–7. 24106351 10.4103/0970-9185.117045 PMC3788225

[4] Dualé C Frey C Bolandard F Epidural versus intrathecal morphine for postoperative analgesia after Caesarean section. Br J Anaesth 2003;91:690–4. 14570792 10.1093/bja/aeg249

[5] Yeh HM Chen LK Lin CJ Prophylactic intravenous ondansetron reduces the incidence of intrathecal morphine-induced pruritus in patients undergoing cesarean delivery. Anesth Analg 2000;91:172–5. 10866907 10.1097/00000539-200007000-00032

[6] Singh SI Rehou S Marmai KL The efficacy of 2 doses of epidural morphine for postcesarean delivery analgesia: a randomized noninferiority trial. Anesth Analg 2013;117:677–85. 23921652 10.1213/ANE.0b013e31829cfd21

[7] Chen MK Chau SW Shen YC Dose-dependent attenuation of intravenous nalbuphine on epidural morphine-induced pruritus and analgesia after cesarean delivery. Kaohsiung J Med Sci 2014;30:248–53. 24751388 10.1016/j.kjms.2014.01.001 PMC11916864

[8] ASA Physical Status Classification System, October 2014. Available at: https://www.asahq.org/resources/clinicalinformation/asa-physical-status-classification-system [access date June 14, 2019].

[9] Sng BL Kwok SC Mathur D Comparison of epidural oxycodone and epidural morphine for post-caesarean section analgesia: a randomised controlled trial. Indian J Anaesth 2016;60:187–93. 27053782 10.4103/0019-5049.177877 PMC4800935

[10] Sultan P Halpern SH Pushpanathan E The effect of intrathecal morphine dose on outcomes after elective cesarean delivery: a meta-analysis. Anesth Analg 2016;123:154–64. 27089000 10.1213/ANE.0000000000001255

[11] Williamson A Hoggart B Pain: a review of three commonly used pain rating scales. J Clin Nurs 2005;14:798–804. 16000093 10.1111/j.1365-2702.2005.01121.x

[12] Hirabayashi M Doi K Imamachi N Prophylactic pentazocine reduces the incidence of pruritus after cesarean delivery under spinal anesthesia with opioids: a prospective randomized clinical trial. Anesth Analg 2017;124:1930–4. 28448397 10.1213/ANE.0000000000002060

[13] George RB Allen TK Habib AS Serotonin receptor antagonists for the prevention and treatment of pruritus, nausea, and vomiting in women undergoing cesarean delivery with intrathecal morphine: a systematic review and meta-analysis. Anesth Analg 2009;109:174–82. 19535708 10.1213/ane.0b013e3181a45a6b

[14] Prin M Guglielminotti J Moitra V Prophylactic ondansetron for the prevention of intrathecal fentanyl- or sufentanil-mediated pruritus: a meta-analysis of randomized trials. Anesth Analg 2016;122:402–9. 26505578 10.1213/ANE.0000000000001046

[15] Aly M Ibrahim A Farrag W Pruritus after intrathecal morphine for cesarean delivery: incidence, severity and its relation to serum serotonin level. Int J Obstet Anesth 2018;35:52–6. 29544720 10.1016/j.ijoa.2018.02.004

[16] Luo J Feng J Liu S Molecular and cellular mechanisms that initiate pain and itch. Cell Mol Life Sci 2015;72:3201–23. 25894692 10.1007/s00018-015-1904-4 PMC4534341

[17] Mo Y Qiu S Effects of dexmedetomidine in reducing post-cesarean adverse reactions. Exp Ther Med 2017;14:2036–9. 28962122 10.3892/etm.2017.4759 PMC5609173

[18] El Aish KA Tafish R Zourob H Morphine versus fentanyl for spinal post-caesarean analgesia: a randomised controlled trial. Lancet 2018;391 Suppl 2:S20. 29553418 10.1016/S0140-6736(18)30386-6

[19] Tsai FF Fan SZ Yang YM Human opioid (-receptor A118G polymorphism may protect against central pruritus by epidural morphine for post-cesarean analgesia. Acta Anaesthesiol Scand 2010;54:1265–9. 21039348 10.1111/j.1399-6576.2010.02310.x

[20] Pettini E Micaglio M Bitossi U Influence of OPRM1 polymorphism on postoperative pain after intrathecal morphine administration in Italian patients undergoing elective cesarean section. Clin J Pain 2018;34:178–81. 28591085 10.1097/AJP.0000000000000520

